# Systems Neuroscience 2021 Top Papers: An Editorial Summary

**DOI:** 10.3390/brainsci12121701

**Published:** 2022-12-12

**Authors:** Konstantin V. Slavin

**Affiliations:** 1 Department of Neurosurgery, University of Illinois at Chicago, Chicago, IL 60612, USA; kslavin@uic.edu; 2 Neurology Service, Jesse Brown Veterans Administration Hospital, Chicago, IL 60612, USA

Many years ago, before the Internet and the introduction of the electronic publications, bibliographical research was conducted in physical libraries, and the most commonly used source of information was the regularly updated Index Medicus, a multi-volume treatise that for 125 years summarized and indexed all published medical literature, classifying it by keywords and subject headings. And whenever researchers or clinicians needed to read the indexed article that was not available in their library, they would mail the corresponding author a request for a reprint. Academic courtesy has routinely been shown by sending paper reprints to all those who requested them, regardless of where in the world such requests would come from. I remember both sending reprint requests when I was writing my first papers and then sending reprints of my own papers in response to such requests; the number of such requests seemed to indicate the level of interest in one’s publications among worldwide readership.

Even now, from time to time, I receive requests for my published papers, not by mail anymore but by e-mail, and the request would be not for a paper reprint but for an electronic file of my article or chapter. The need for this process was essentially obviated by the concept of “open access” that allows any interested party to instantly retrieve published articles free of charge. Moreover, with widespread electronic publishing and indexing, the interest to, relevance and impact of one’s publications are no longer measured by the number of reprint requests, but rather by the number of citations and downloads. These indirect measures are now easy to track, and our journal’s publisher, MDPI, recently put together a list of the top ten articles in the section “System Neuroscience” published in 2021 that ranked highest in citations and “view and download” over the subsequent year. Below is the brief summary of these ten papers; its purpose is to further raise awareness of the most read topics and increase visibility of the high level of research covered in this section.

The somewhat eclectic nature of the discussed papers is not surprising as the section “Systems Neuroscience” has probably the widest spectrum of covered topics. Six reviews and four research articles made the “Top Ten” list of 2021.

A thorough review of congenital disorders of glycosylation by Paprocka et al. [1] focuses on neurological aspect of this rare but remarkably devastating group of metabolic abnormalities. Incorrect glycosylation of proteins is known to disrupt the function of many organs and tissues, which results in complex systemic pathology and clinical symptomatology. As noted by the authors, phenotypes and severity of clinical presentations vary significantly, but most patients exhibit severe neurological dysfunction, including epileptic seizures, intellectual disability, myopathy, neuropathy, ataxia and stroke-like episodes. The review summarizes a variety of published neurological manifestations and congenital brain malformations and matches them with different subtypes of disorders of glycosylation.

Another review paper is dedicated to stressful and potentially detrimental effects of extremely low frequency (ELF) magnetic fields [2]. Researchers from Copernicus University in Poland critically analyzed the literature data on biological interactions caused by exposure to ELF magnetic fields. These fields, with frequencies between 0 and 300 Hz, are widespread in the modern environment and come from natural and man-made sources; the ubiquitous exposure to ELF magnetic fields is thought to be associated with changes in lipid profile and calcium metabolism, protein levels and oxidative stress, pro-inflammatory cytokines and neurotrophic factors. Not surprisingly, such a stressor has been linked to increased incidence of depression, anxiety, sleep disorders and impairment of emotional behavior and wellbeing. However, in addition to the negative effects, there is a possibility of positive influence through stimulation of brain plasticity and increase in the activity of antioxidants. All of this is summarized by Klimek and Rogalska in a systematic fashion, with the goals of better understanding the observed phenomena, a reduction in associated risks and the recognition of possible therapeutic implications. 

Next among the top ten papers is the comprehensive review of oxidative dysregulation in early life stress and post-traumatic stress disorder (PTSD) by Karanikas et al. [3]. The authors attempted to analyze the connection between traumatic stress and oxidative pathways, homeostasis and redox-state, covering “clinical, genetic and epigenetic human findings on trauma-related oxidative stress” and discussing these findings in the context of systemic inflammation, the neuro-endocrine axis and the circadian system. The high complexity of neurobiological mechanisms and multi-dimensional interactions between traumatic stress and oxidative dysregulation prompts an encouragement for researchers to focus on underlying links and substrates, potentially enhancing future therapeutic and preventive strategies for exposed individuals.

An investigation of the relationship between basic cognitive functions and sport-specific physical performance in young (10–12 year old) volleyball players is a focus of research study by Trecroci et al. from several Italian universities [4]. Based on the analysis of 43 study participants, the study authors found that in addition to large positive correlations between cognitive functions and sport-specific physical performance, there were correlations between the athletes’ cognitive and motor skills. The findings were in line with previous data on soccer players, supporting the general concept of an existence of structural and functional links between neural networks responsible for cognitive and motor tasks and possibly helping in scouting talented sportsmen through a multidisciplinary process.

An inherent challenge for the delivery and distribution of drugs in the central nervous system and the inner ear—mainly related to the existence of the blood–brain and blood–perilymph barriers—may be addressed with the use of superparamagnetic iron oxide nanoparticles (SPIONs). A systematic literature review by Guigou et al. [5] yielded 97 studies on this topic published between 1999 and 2011. In a well-illustrated and well-referenced format, the authors analyzed the current state-of-the-art in this field, reviewed different SPIONs concepts and characteristics, listed potential indications and toxicity and concluded with the need to conduct further studies on specific nuances in this promising direction.

The current lack of clear understanding about the mechanism by which transcranial alternating current stimulation (tACS) modulates endogenous oscillations in the human brain prompted Chen et al. [6] to conduct a graph theoretical functional MRI study on 13 chronic stroke patients. Using 10 Hz and 20 Hz tACS, the researchers analyzed the effects of stimulation on network integration and segregation. Observed effects were frequency-dependent, and some characteristics changed in opposite directions with stimulation at different frequencies. tACS at 20 Hz was found to induce more modulation effects, suggesting a higher potential of its applicability in rehabilitation therapies.

Industrial assembly today is remarkably different from its original concept when assembly workers had to repeatedly and routinely assemble homogeneous products. Nowadays, increasing customization in the assembly process leads to a much higher degree of complexity when heterogeneous product variants are created through so-called mixed model assembly. This, in turn, results in a significant increase in mental workload as each episode of information processing is associated with elements of choice, uncertainty and randomness. To investigate the influence of complexity and use of informational assistance systems on mental workload, Bläsing and Bornewasser studied physiological responses and performance indicators in 65 research subjects using simulated assembly tasks of different complexities with or without three different assistance systems [7]. Task complexity influenced the degree of the mental workload, but, somewhat surprisingly, the assistance systems had no independent beneficial or reducing effect on the mental workload as performance results were solely determined by the quality of instructions. The presented data have to be taken into consideration when a program of “cognitive neuroergonomics” is being developed and implemented.

A fascinating anatomical structure designated as the “blue spot” (in Latin—locus coeruleus) is a pigmented structure of the rostral hindbrain that contains noradrenaline-producing neurons. Due to the consistent presence of the locus coeruleus in mammalian species, scientific findings in laboratory animals have been extrapolated to the human physiology—but the inter-species variability has not been systematically analyzed. Manger and Eschenko reviewed the available information from approximately 80 mammalian species across different subclasses and orders and created a comparative analysis of the nuclear organization of the locus coeruleus complex [8]. The described variations in nuclear organization have to be accounted for in the cross-species conclusions and practical implications, as the potential homology may or may not be correct.

Mechanical vibration in the workplace is known to produce a variety of harmful effects, but recent studies elucidated positive effects of whole-body vibration on different body systems, including the potential change in synaptic plasticity. To investigate the modulation of synaptic plasticity by vibratory training, Cariati et al. applied three different protocols (varying by frequency and exposure time of the vibrations) in groups of mice of different ages (4 and 24 months old) and then performed extracellular recording in hippocampal slices [9]. Results of this research study demonstrate not only the presence of modulation of synaptic plasticity by vibratory training but also the differences in results based on vibration frequency and the exposure time to vibration. Moreover, the authors suggest that the effects of vibration training on cognitive processes may vary with age—information that may be useful in preventing the development of age-related cognitive impairments.

Biggio et al. authored an in-depth review of the recent basic science and clinical research of melatonin (N-acetyl-5-methoxytryptamine), a chemical substance that serves as a major regulator of the mammalian sleep/wake cycle [10]. They summarized published data on “multifactorial effects of endogenous and exogenous melatonin on different physiological (pregnancy, brain development, and aging), pharmacological (action through specific receptor complexes) and pathological (sleep disturbances and other diseases) conditions”. Based on the literature analysis, the authors were able to demonstrate the great chronobiotic efficacy and sleep-promoting effects of exogenous melatonin. Moreover, the potential ability of melatonin to facilitate neuronal resilience to stress may become a direction for future clinical applications.

Hopefully, this brief summary of the top 10 most viewed and downloaded articles published in 2021 in the section of “Systems Neuroscience” will prompt the reader to learn more about each of these ten fascinating topics. There are literally hundreds of high-quality peer-reviewed papers published in our section and others of the *Brain Sciences* journal every year, with each being immediately available to the worldwide audience free-of-charge thanks to the open access publication model. This editorial is aimed at raising awareness of the diverse spectrum of topics covered by our authors and contributors.
